# A curious interplay in the films of N-heterocyclic carbene Pt^II^ complexes upon deposition of alkali metals

**DOI:** 10.1038/srep25548

**Published:** 2016-05-06

**Authors:** Anna A. Makarova, Elena V. Grachova, Dorota Niedzialek, Anastasia I. Solomatina, Simon Sonntag, Alexander V. Fedorov, Oleg Yu. Vilkov, Vera S. Neudachina, Clemens Laubschat, Sergey P. Tunik, Denis V. Vyalikh

**Affiliations:** 1 Institut für Festkörperphysik, Technische Universität Dresden, 01062 Dresden, Germany; 2 Institute of Chemistry, St. Petersburg State University, 198504 St. Petersburg, Russian Federation; 3 Department of Physics, Centre for Plastic Electronics, Imperial College London, SW7 2AZ London, UK; 4 Leibniz-Institut für Festkörper- und Werkstoffforschung Dresden, 01171 Dresden, Germany; 5 II Physikalisches Institut, Universität zu Köln, 50937 Köln, Germany; 6 Department of Physics, St. Petersburg State University, 198504 St. Petersburg, Russian Federation; 7 Department of Chemistry, Moscow State University, 199991 Moscow, Russian Federation

## Abstract

The recently synthesized series of Pt^II^ complexes containing cyclometallating (phenylpyridine or benzoquinoline) and N-heterocyclic carbene ligands possess intriguing structures, topologies, and light emitting properties. Here, we report curious physicochemical interactions between *in situ* PVD-grown films of a typical representative of the aforementioned Pt^II^ complex compounds and Li, Na, K and Cs atoms. Based on a combination of detailed core-level photoelectron spectroscopy and quantum-chemical calculations at the density functional theory level, we found that the deposition of alkali atoms onto the molecular film leads to unusual redistribution of electron density: essential modification of nitrogen sites, reduction of the coordination Pt^II^ centre to Pt^0^ and decrease of electron density on the bromine atoms. A possible explanation for this is formation of a supramolecular system “Pt complex-alkali metal ion”; the latter is supported by restoration of the system to the initial state upon subsequent oxygen treatment. The discovered properties highlight a considerable potential of the Pt^II^ complexes for a variety of biomedical, sensing, chemical, and electronic applications.

Within the past decades, a lot of attention has been paid to the investigation of molecular organic semiconductors (OSCs) to be used in various optical and electronic devices, including organic light-emitting diodes (OLEDs), organic field effect transistors (OFETs), organic photovoltaic cells (OPVCs), and organic spintronic devices^1^,^2^,^3^,^4^. Metal doped organic materials have been of particular interest due to their unique magnetic^5^, electronic^6^,^7^, and physical properties^8^, which are unachievable in pure organic compounds. For example, it was shown that OSCs doped with transition metals are able to display room temperature ferromagnetism due to bound magnetic polarons^5^. In turn, doping via intercalation of alkali metals has proven to be one of the most efficient methods to overcome the inherent limitations of pure OSCs: to decrease the barrier height for carrier injection at interfaces in OLEDs, to enhance the electron conductivity of materials; and, therefore, to improve the performance of the devices^9^,^10^,^11^,^12^. Both pure and metal-doped structures have been studied comprehensively using, *inter alia*, photoemission spectroscopy^13^, and for most structures either charge transfer or chemical interaction between the metal and the substrate were reported. Phthalocyanines (Pcs)/metal-Pcs and perylenetetracarboxylic dianhydride (PTCDA) have probably become the most thoroughly investigated compounds of this type due to their attractive electronic properties. Pcs/metal-Pcs and PTCDA are planar molecules with a high degree of symmetry and can be easily deposited in ultra-high vacuum (UHV) with no changes in their structure^14^,^15^. Representatives of another group of OSCs, namely non-planar organic molecules with a high degree of symmetry (e.g. a PTCDA derivative, DiMe-PTCDI^15^, and Tris(8-hydroxyquinolinato)aluminium (Alq_3_)^12^,^16^,^17^) have been a subject of numerous studies as well. In contrast, information on the possibility to form ordered systems based on non-planar molecular OSCs with low symmetry in UHV, as well as on their doping with alkali metals remains scarce, although from the chemical and electronic viewpoints such systems are of particular interest, as specified below.

Recently a new generation of cyclometallated platinum complexes with tunable light emitting properties and low symmetry of the coordination sphere has been synthesized and characterized^18^,^19^,^20^,^21^,^22^. A family of [Pt(N^C)(NHC)L] complexes with coordinated: (i) cyclometallating N^C ligand (2-phenylpyridine or 7,8-benzoquinoline), (ii) N-heterocyclic carbene (NHC) (1,3-dibenzylbenzimidazolium, 1,3-diethylbenzimidazolium or 1,3-dibenzylimidazolium), and (iii) Cl, Br or C_2_Ph as the L moiety is of particular importance^23^. These compounds are organic semiconductors that demonstrated high stability in various media, and can be converted into molecular films under UHV conditions by physical vapour deposition (PVD). They also display tunable light emitting properties, which make them extremely attractive for applications in phosphorescence organic light emitting devices, biomedical and sensing technologies. Note that the solid state structures of these complexes^23^ are almost identical and display a square-planar geometry with the N^C cyclometallating ligand coordinated in a chelated manner to form five-membered metallocycles (see Fig. 1a and SI, Fig. S1). The NHC ligand occupies the *trans*-position with respect to the nitrogen atom of the metallocycle and is not coplanar with the platinum centred quadrangle. This geometry results in a lack of delocalization of the electronic density over the whole molecule^23^, which is remarkably different from the Pcs complexes and other organic molecules with π-conjugation, for which chemical interactions with alkali metal atoms have been actively studied within the past decade^24^,^25^,^26^,^27^,^28^,^29^,^30^,^31^.

In the present study we focused on one typical representative of the described Pt^II^ compounds, [Pt(N^C)(NHC)Br] (See Fig. 1a for its structure). First, we describe the deposition of thin films under UHV and characterize them as-grown. Further on, such samples were used to explore comprehensively how deposited alkali metal atoms interact with the molecules of the platinum complex in the films and what the partners in this interaction are. The subjects of particular interest were: (i) how the redistribution of electronic density occurs, (ii) how close the metal atoms can approach to the Pt^II^ centre, and (iii) how they can couple to it. It should be noted that all representatives of the family of [Pt(N^C)(NHC)L] complexes were tested, and the results demonstrate a certain general trend in interaction with alkali metal atoms (see Fig. S2 for illustrations using certain metal-complex pairs). We believe that the thorough insight into the electronic and chemical structure of Pt^II^ complexes modified by alkali metals, as presented here, will pave the way towards their novel applications and will support further technological development of these fascinating materials.

## Results and Discussion

### Growth of the Pt^II^ complex film in UHV

First, we applied the well-known PVD approach for growing high quality molecular layers on solid state substrates to obtain the [Pt(N^C)(NHC)Br] complex film on Au(111) under UHV conditions.

**Figure 1:**
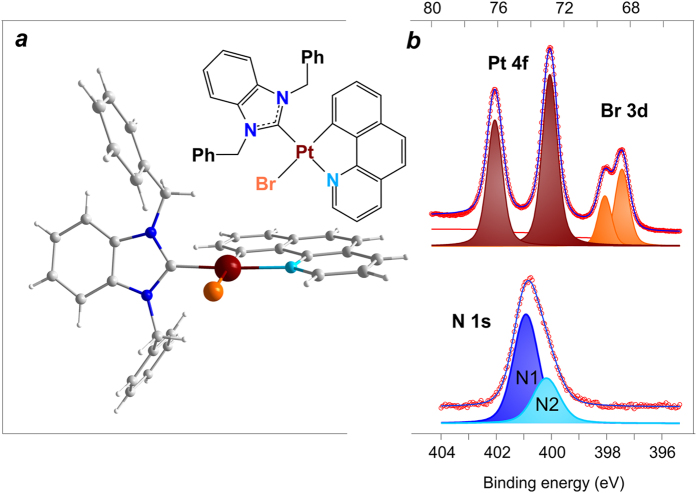
(**a**) Schematic representation (top) and the molecular structure (bottom) of the Pt complex under investigation. (**b**) Photoemission core-level N 1s, Pt 4f and Br 3d spectra taken from freshly deposited molecular layers of the Pt complex on Au substrate. Color legend for the molecular state structure: platinum brown, bromine orange, nitrogen blue/light blue, carbon grey, hydrogen white.

To evaluate the quality and to control the stoichiometry of the *in situ* grown molecular film, photoemission (PE) spectra of the N 1s, Pt 4f and Br 3d core levels were collected. A typical set of spectra is shown in Fig. 1b. The N 1s line can be fitted with 2 singlet peaks arising from different chemical states of the nitrogen atoms. This is because the system has two types of nitrogen atoms (*N1* and *N2*) in the NHC and N^C ligands *trans*-located in the Pt^II^ coordination sphere. The intensity ratio *N1*:*N2* is approximately 2:1. The *N1* feature with a binding energy (BE) of 400.9 eV corresponds to nitrogen atoms in the NHC ligand^32^, while the component *N2* at 400.2 eV can be assigned to the electron emission from the nitrogen atoms in the N^C ligand. Pt 4f and Br 3d are spin-orbit split doublets corresponding to a single chemical state of the atoms. Detailed analysis of the Pt 4f spectrum allows concluding that the 4f_7/2_ component at BE 72.8 eV corresponds to the Pt^II^ oxidation state^33^; the Br 3d_5/2_ peak detected at BE of 68.5 eV is due to the bromide ligand. All these observations conclusively indicate that the molecular complex remains unmodified upon deposition onto the metallic substrate. Using the deposited films, we further aimed at modifying their electronic and chemical structure by *in situ* deposited alkali metals.

### Doping of the Pt^II^ complex with alkali metals

#### Photoelectron spectroscopy of core-levels

**Figure 2:**
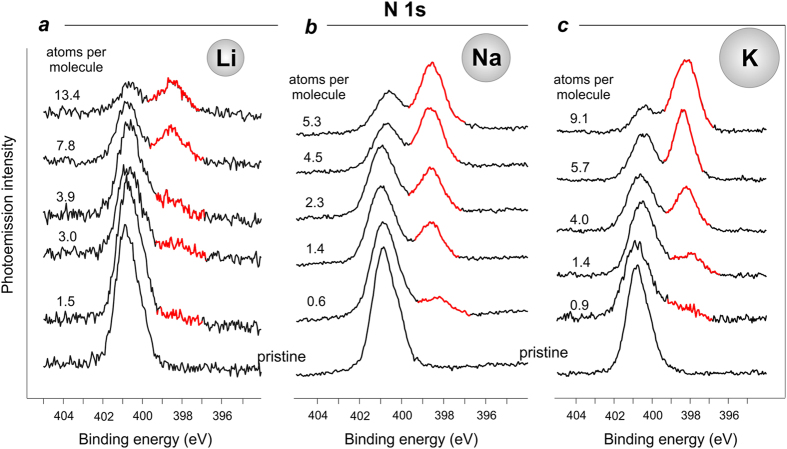
N 1s core-level PE spectra of the pristine Pt(II) complex thin film, and the same film after gradual deposition of Li (**a**), Na (**b**), and K (**c**) on top.

For our doping experiments, we used Li, Na, K, and Cs and investigated the similarities and differences in their interplay with the Pt^II^ molecular complex. Figure 2 shows the evolution of the N 1s core-level spectrum as Li, Na and K were gradually deposited on top of the Pt^II^ complex film. A brief inspection suggests that there is a certain general trend in the modification of the spectral lineshapes for all three studied systems; a new spectral feature appears at lower BEs compared to the main peaks, while the intensity of the native structures decreases gradually. The observed spectral changes apparently indicate that the nitrogen sites of the molecule are attacked by the deposited alkali atoms, and charge is transferred to the nitrogen sites.

**Figure 3:**
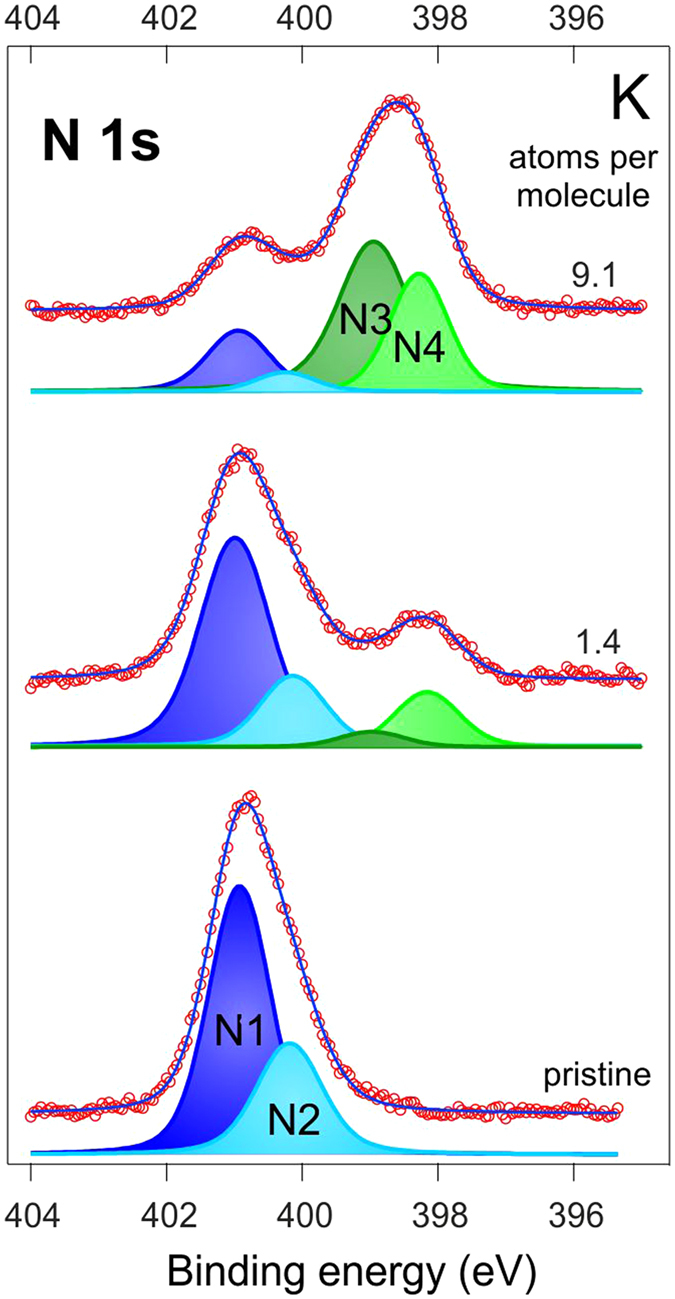
N 1s PE spectra and their decomposition for the pristine sample (bottom) and after K deposition of concentrations of 1.4 (middle) and 9.1 atoms per molecule (top).

To understand which of the two nitrogen atoms becomes modified by the deposition, we fitted the spectra obtained in the experiments. Let us consider the results by the example of potassium, see Fig. 3. K deposition onto a freshly prepared molecular layer results in an intensity decrease of the original components, *N1* and *N2*, which are gradually transforming into the new *N3* and *N4* peaks, respectively. It is noteworthy that the first stage of the process when the concentration of potassium amounts to about 1.4 atoms per complex molecule the *N2* peak seems to be more affected, which indicates higher potassium affinity to the *N2* nitrogen site (N^C cyclometallating ligand) at this stage. Upon further potassium deposition the intensity of the *N3* feature grows more rapidly, which indicates further effective involvement of NHC-ligands into the interaction process. Therefore, we conclude that both nitrogen sites are affected by the interaction between potassium and the molecular film.

**Figure 4:**
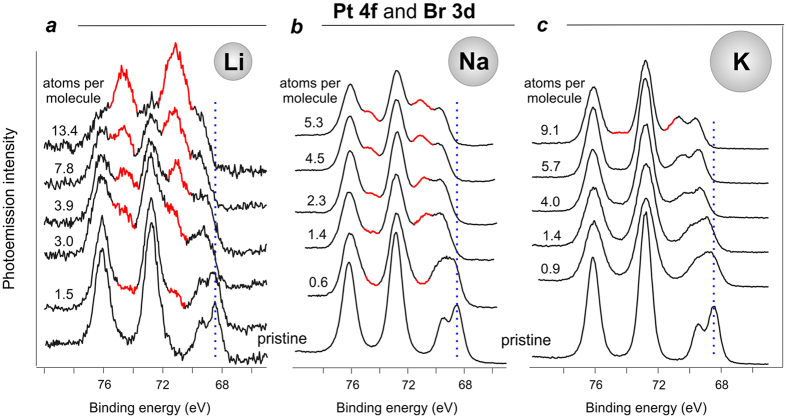
Overlapping Pt 4f and Br 3d core-level PE spectra of the pristine Pt(II) complex thin film, and the same sample after gradual deposition of Li (**a**), Na (**b**), and K (**c**).

Further insight into the chemical modifications of the Pt^II^ complex in the presence of alkali metals has been obtained from the analysis of the Pt 4f and Br 3d core levels, which are shown in Fig. 4. The presented sequences of PE spectra were taken together with those given in Fig. 2. It is clearly seen that deposition of alkali metal atoms led to the appearance of a new spectral feature in the Pt 4f spectra, which is centred at 71.4 eV. The intensity of this new peak, which can be attributed to chemically reduced Pt-centre (Pt^2+^ to Pt^0^)^33^, decreases with the atomic number of the deposited alkali element (*i.e*. in the order: Li, Na, K, Cs, see Figs 4 and S3). A joint consideration of both Pt 4f and N 1s spectra (Figs 2 and 4) suggests that Li incorporation (*i.e*. that of the smallest alkali atom) affects the Pt-centre in a more pronounced manner than the N-sites of the complex. Due to their small size and high mobility, lithium atoms can easily approach the molecule centre and attack the positively charged Pt, thus reducing it from Pt^II^ to Pt^0^.

The increase in the atomic radius for the alkali metal atom obviously decreases its ability to approach the molecule centre within the film due to the sterical factor, and consequently less Pt^II^ centres are reduced to the Pt^0^ state. Therefore, bigger alkali atoms interact presumably with the sterically available N-sites. This trend is confirmed by further analysis of the available spectral data (see Figs 2 and 4): for the concentrations of deposited alkali metal close to 4 atoms per molecule (Li-3.9, Na-4.5, K-4.0) the percentage of the reduced Pt-centres was 43.9 for Li, 29.7 for Na, and 8.1 for K, while the percentage of the modified N-sites was 17.0, 55.0, and 39.8 (as compared to the overall intensity of the N 1s line) for Li, Na and K, respectively. Although the positive charge on platinum makes it a good target for interactions with the alkali metal atoms, the bulky ligands sterically shield the Pt^II^ ions from the nucleophile attack.

At the first glance, considerable increase in the electron density localized at the nitrogen and platinum sites clearly points to charge transfer from the alkali metal atoms to the complex molecule resulting in formation of an ion pair: radical anion and positive counterion M^+^ (where M = Li, Na, K, Cs). Such behaviour is common for the systems containing alkali metals and π-conjugated organic materials^34^. However, it is hard to describe the system behaviour in terms of a simple platinum complex reduction^17^,^35^ taking into account the spectroscopic data for the bromide ligand. In the course of Li, Na, K, and Cs deposition on top of the film formed by the Pt^II^ complex we observed a gradual shift of the Br 3d spectral feature towards higher BEs simultaneously with the increase in the alkali metal concentration (see Fig. 4 and Fig. S3b in SI). In contrast to the nitrogen and platinum centres, the bromide ligand loses electron density to serve as an electron donor.

This seems to be rather natural since bromide possesses four labile electron pairs and is known to act only as a π- and σ-donor. These observations can be rationalized by the “cation-anion pair” interaction between the alkali metal cation and the bromide anion, which occurs due to oxidation of the metal atom to give further redistribution of electron density. In contrast to the N 1s and Pt 4f spectral series, which are characterized by multicomponent structures with original and new features changing their relative intensities upon alkali metal incorporation, the Br 3d spectra demonstrate a gradual shift of the lines reflecting a gradual decrease of the electron density on the Br^−^ anion. This tendency implies that one bromine ion can interact with several alkali metal ions. With the increasing amount of alkali atoms incorporated into the molecular layer, the number of electrostatic interactions between the metal ions and a certain bromine atom should also increase, thus resulting in the shifts of the Br 3d peak towards higher BEs. Presumably, during the deposition, the gradually increasing number of alkali metal atoms surrounding the [Pt(N^C)(NHC)Br] molecules leads to the formation of a supramolecular structure within the molecular film, which is held together by a network of electrostatic interactions between the alkali cations and bromide ligands in the platinum complex.

Additionally, the deposited alkali metal atoms also modify the electron density on other atoms in the peripheral part of the ligands. For example, in the C 1s core-level spectra (see Fig. S4 in SI), we observe a significant line broadening together with the formation of a shoulder at lower BEs upon deposition of alkali metal. Therefore, we suggest that alkali metal atoms affect to some extent the aromatic systems^36^ of the NHC and N^C ligands either by direct interaction with the peripheral sites or as a result of molecular topology distortion.

#### Calculations

**Figure 5:**
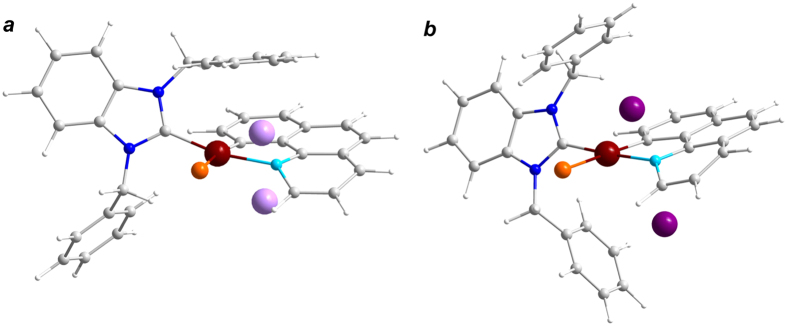
DFT-optimized molecular structure of [Pt(N^C)(NHC)Br] complex with 2 incorporated alkali metal atoms: Li (**a**), K (**b**). Color legend: platinum brown, bromine orange, nitrogen blue/light blue, carbon grey, hydrogen white, lithium lavender; potassium purpura.

In order to determine the most favorable sites occupied by alkali metals in the vicinity of each [Pt(N^C)(NHC)Br] molecule we have performed a series of DFT calculations. The starting positions of the alkali metal atoms for the optimization have been assigned based on our estimations obtained from the X-ray photoelectron spectroscopy (XPS) data, *i.e*. ca. 3 Å above the Pt-N1 axis. Then, we moved the starting position of the alkali metals by + /− 0.5 Å along that axis in order to achieve a better description. In all cases under study, we observed adjustments of the alkali metal atom positions towards the Pt-centre upon structure optimization (see Fig. 5). As expected, the larger the van der Waals radius of the alkali metal atom, the bigger the distance between it and the Pt-centre (see Table 1).

Moreover, we performed Mulliken population analysis of these optimized structures to estimate the extent of electron redistribution in the [Pt(N^C)(NHC)Br] molecule upon interaction of an isolated molecule with different alkali metals. We observed substantial charge redistribution in the presence of the alkali metals (see SI, Figs S5–S7), *i.e*. reduction of the Pt-centre. It should be noted that the simulated systems consisted of one isolated [Pt(N^C)(NHC)Br] molecule, which made the approach of all alkali metal atoms (independent of their size) to the Pt-centre and its reduction quite possible, while experimental results show that effective reduction of the Pt centre is only possible for the smaller alkali metals (*e.g*. Li and Na). This inconsistency with the experimental data can be explained by densely packed geometry of the molecular film. Smaller alkali atoms may not only come closer to the platinum, but also their number around the Pt centre can be higher, leading to even stronger charge transfer to the Pt centre. Alkali metal atoms are also involved in charge transfer interaction with the N2-atoms (of N^C cyclometallating ligand), which lead to an increase in the electron density localized on them and a noticeable (~1.2–1.4 Å) decrease of the Pt-N bond length (*i.e*. to essential growth of double bond character of this bond due to the presence of additional electronic density at the nitrogen site) (see Table 1). Moreover, the interaction between the N2-atom and the alkali atoms led in almost all cases to deformation of the N^C-ligand.

Once ionized, *e.g*. because of the interactions with the Pt-centre and/or with the N2-atom, the alkali metal atom is positively charged and thus can interact with the bromide ion to provoke electron density withdrawal from the latter (see Figs S5–S7), as it was shown by the spectroscopic data.

### Pt^II^ complex recovery under oxygen treatment

The model describing the formation of the supramolecular system via interaction of alkali metals with the film of the Pt^II^ complex fits well the data obtained using N 1s, Pt 4f and Br 3d core-level photoemission spectra. However, there are certain concerns regarding the non-destructive character of such interaction. Since the BE of the newly formed Pt 4f feature corresponds to that of Pt^0^ (see Fig. 4), it could be suggested that the complex undergoes degradation upon interaction with alkali metals that apparently leads to Pt aggregation into metal nanoclusters.

To address this issue, we have performed an additional experiment featuring the system recovery in the oxygen atmosphere. In this case, we expected that oxygen, as an electron acceptor, may withdraw the charge from the supramolecular system “Pt complex-alkali metal ion”, thus recovering the initial platinum complex.

**Figure 6:**
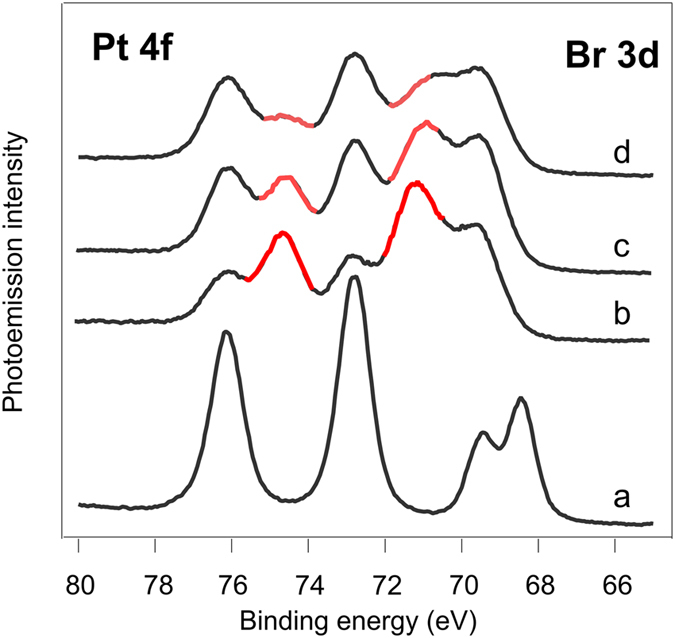
Set of overlapping Pt 4f and Br 3d spectra for the pristine Pt(N^C)(NHC)Br complex (**a**), after deposition of ca. 8 Li atoms per complex molecule (**b**), and after two steps of oxygen exposure under 10^−5^ mbar: up to 2.3 × 10^4^ L (**c**) and up to 5.7 × 10^4^ L (d).

The most pronounced changes were observed in the Pt 4f spectra for the system with Li, which are shown in Fig. 6. The spectra taken after exposure of the system to oxygen reveal partial oxidation of the formerly reduced platinum: significant decrease of the Pt^0^ component intensity, and at the same time enhancement of the original peak corresponding to Pt^II^ ions. Therefore, we can confirm our conclusion, namely, that no complete platinum reduction Pt^II^ → Pt^0^ leading to destruction of the complex and agglomeration of metallic nanoparticles takes place; we rather observe a reversible process of electron density redistribution. This scenario is also supported by the analysis of the respective N 1s spectra (not shown here). The observed effect of the original complex recovery is well-known as the effect of n-doping compensated by p-type dopants^26^,^37^.

Finally, we interpret the results of the spectroscopic measurements and theoretical calculations as follows:

- Alkali metals have a strong affinity towards the positively charged Pt^II^-centre of the [Pt(N^C)(NHC)Br] complex;
- Nevertheless, for alkali metal atoms with smaller atomic radius (*e.g*. Li, Na) it is easier to come close to the Pt-centre and consequently reduce Pt^II^ to Pt^0^ due to a large steric hindrance arising from the molecular topology and densely packed geometry of the molecular film. At the same time, electron density is also transferred to the two nitrogen sites;
- Larger alkali metal atoms that cannot reach the Pt centre as easy as the smaller ones can still approach and interact with the ligands, i.e. modify the electronic density on the nitrogen and carbon atoms;
- Once an alkali atom is ionized it tends to interact with bromide ions electrostatically;
- Under exposure of the doped system to oxygen the “Pt complex-alkali metal ion” structure is able to partly recover to give the initial Pt^II^ complex.

## Conclusion

In summary, the recently synthesized Pt^II^ complexes [Pt(N^C)(NHC)L] containing cyclometalated N^C and N-heterocyclic carbene ligands have been successfully deposited under UHV conditions on a Au(111) substrate to form a molecular film. By performing *in situ* deposition of Li, Na, K and Cs on its surface, we have demonstrated an intriguing chemistry of these systems. Both experimental data and theoretical calculations show that alkali metal atoms interact with the molecular film and attach directly to the positively charged Pt-centre. However, the size of the alkali metal atoms has a significant effect on their final position with respect to the Pt-centre and the quantity of the reduced Pt atoms. The smallest and highly mobile Li atom can easier reach the Pt^II^ centre, thus reducing it to Pt^0^. This route is hindered for large alkali atoms. As their approach towards the Pt atom is halted, they only slightly affect the latter. Instead, these larger metals share their electrons more effectively with the orbitals located around the N-sites of the ligands, leading to distortion of these peripheries of the complex. Finally, our results suggest a presence of electrostatic interactions between the Br-site of the Pt-complex and the alkali metal ions surrounding it. This remarkable behaviour of the studied platinum complex allows concluding that the final system is not a simple ion-pair complex but a supramolecular ‘Pt complex-alkali metal ion’ structure, held together by a network of electrostatic interactions. Interestingly, treating the “Pt-complex-alkali metal ion” system with oxygen led to its recovery towards the pristine Pt^II^-complex. We believe that the current study will improve understanding of the nature of the fundamental physicochemical interaction between metal atoms and organic/organometallic materials. We anticipate that this fascinating chemistry involving recently synthesized organometallic films, alkali metals, and technologically important electron doping effects, will result in interesting applications of these systems in a variety of fields.

## Experimental and Calculational Methods

### Sample preparation

[Pt(N^C)(NHC)Br] complex were synthesized and characterized as described previously^23^. Samples of molecular films were prepared using the well-elaborated PVD approach under UHV conditions (at the base pressure of 10^−9^ mbar). Before the deposition the substrate (Au(111) single crystal) surface was cleaned by the conventional procedure of repeated cycles of Ar ion sputtering and annealing in the UHV. No remaining contamination was detected in the corresponding core-level PE spectra. [Pt(N^C)(NHC)Br] in form of powder was introduced into a Ta crucible, which was transferred into a custom-made evaporator in the UHV. In order to avoid any residual contamination the molecular powder was degassed for 3 hours at 140 °C. To perform the evaporation, the source was heated using electron beam. The source temperature was measured by a Tungsten-Rhenium thermocouple in close contact with the crucible. The minimum sublimation temperature that we experimentally found was 230 °C. Upon deposition the substrate was kept at room temperature. No subsequent annealing of the deposited film was performed. The corresponding core-level PE spectra showed that molecular films of [Pt(N^C)(NHC)Br] were grown without any traces of contamination.

The stoichiometry of the deposited films was controlled according to the well-elaborated approach of quantitative X-ray photoelectron spectroscopy measurements. For the doping studies, pure alkali metals were evaporated onto the molecular film from a properly outgassed SAES alkali-metal dispenser. The deposition rate was monitored with a quartz microbalance. The concentrations of alkali atoms in the [Pt(N^C)(NHC)Br] films were evaluated from a comparison of the relative intensities of the Li 1s, Na 2s, K 2p and Pt 4f and/or Br 3d core-level signals.

### Experimental details

All the spectroscopic measurements were performed using synchrotron radiation from the Russian-German Beamline^38^ at BESSY II, Helmholz Zentrum Berlin. Photoemission spectra were acquired with a hemispherical Phoibos 150 electron energy analyzer (SPECS GmbH) for high-energy resolution PE experiments. PE measurements were carried out in the normal emission geometry with photon energies of 520 eV. All the measurements were carried out at room temperature. The spectra obtained were fitted using Gaussian-Lorentian convolution functions with a simultaneous background optimization.

### Calculations

The *ab-initio* optimizations of the structures with and without the alkali ions were performed using the GAUSSIAN09 software^39^. The ligand structures were optimized at the DFT level, using the HSEH1PBE functional^40^ and the 6–31G(d) basis set^41^. For the metal (i.e. Pt-centre and alkali metals) the LANL2DZ basis set was used, as it contains an effective core potential for heavy atoms.

## Additional Information

**How to cite this article**: Makarova, A. A. *et al*. A curious interplay in the films of N-heterocyclic carbene Pt^II^ complexes upon deposition of alkali metals. *Sci. Rep*. **6**, 25548; doi: 10.1038/srep25548 (2016).

## Supplementary Material

Supplementary Information

## Figures and Tables

**Table 1:** DFT-optimized distances between the relevant atoms in the pristine Pt(N2^C)(N1HC)Br molecule and in the complexes with two alkali metals atoms (M = Li, Na, K, Cs).

Distance [Å]	Pt(N^C)(NHC)Br				
	Molecule only	doped with Li	doped with Na	doped with K	doped with Cs
Pt-N2	2.2	2.08	2.07	2.05	2.06
Pt-Br	2.5	2.65	2.67	2.65	2.63
Pt…M	–	2.62–2.97	2.89–2.99	3.20–3.44	3.66–3.85
N2…M	–	1.92–2.07	2.33–2.48	2.66–2.81	3.09–3.23
Br…M	–	2.52–4.15	2.84–4.53	3.32–4.79	3.89–5.13
