# Supplementary material for: A curious interplay in the films of N-heterocyclic carbene PtII complexes upon deposition of alkali metals
Source: Sci Rep. 2016 May 6;6:25548. doi: 10.1038/srep25548 (PMC4858761; doi:10.1038/srep25548)
Supplement: Supplementary Information [file srep25548-s1.pdf]

Supplementary Information

A curious interplay in the films of N-heterocyclic carbene Pt^{II} complexes upon deposition of alkali metals

Anna A. Makarova[1*], Elena V. Grachova[2], Dorota Niedzialek[3], Anastasia I. Solomatina[2], Simon Sonntag[1], Alexander V. Fedorov[4,5,6], Oleg Yu. Vilkov[6], Vera S. Neudachina[7], Clemens Laubschat[1], Sergey P. Tunik[2], and Denis V. Vyalikh[1, 6]

[1]*Institut für Festkörperphysik, Technische Universität Dresden, 01062 Dresden, Germany*

[2]*Institute of Chemistry, St. Petersburg State University, 198504 St. Petersburg, Russian Federation*

[3]*Department of Physics, Centre for Plastic Electronics, Imperial College London, SW7 2AZ London, UK*

[4]*Leibniz-Institut für Festkörper- und Werkstoffforschung Dresden, 01171 Dresden, Germany*

[5]*II Physikalisches Institut, Universität zu Köln, 50937 Köln, Germany*

[6]*Department of Physics, St. Petersburg State University, 198504 St. Petersburg, Russian Federation*

[7]*Department of Chemistry, Moscow State University, 199991 Moscow, Russian Federation*

* *Corresponding author:* aa.makarova@yandex.com

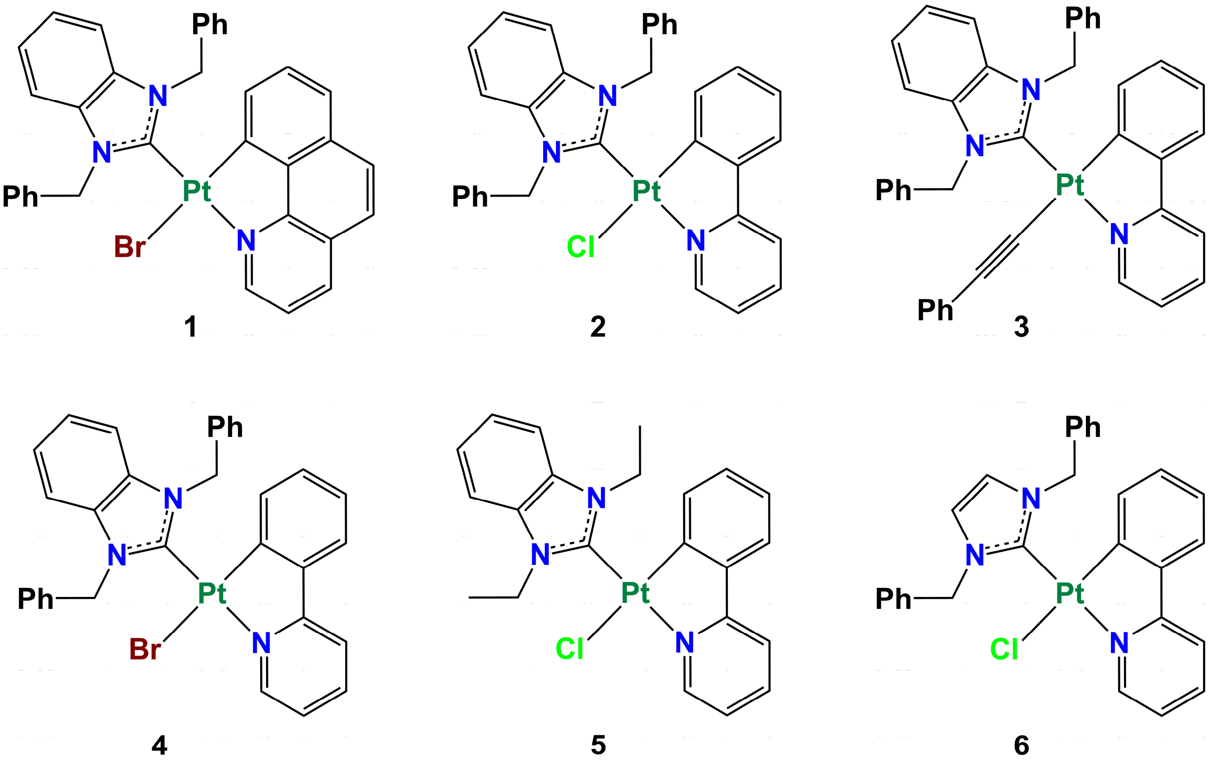

Figure S1. Schematic representation of the [Pt(N^C)(NHC)L] complexes.

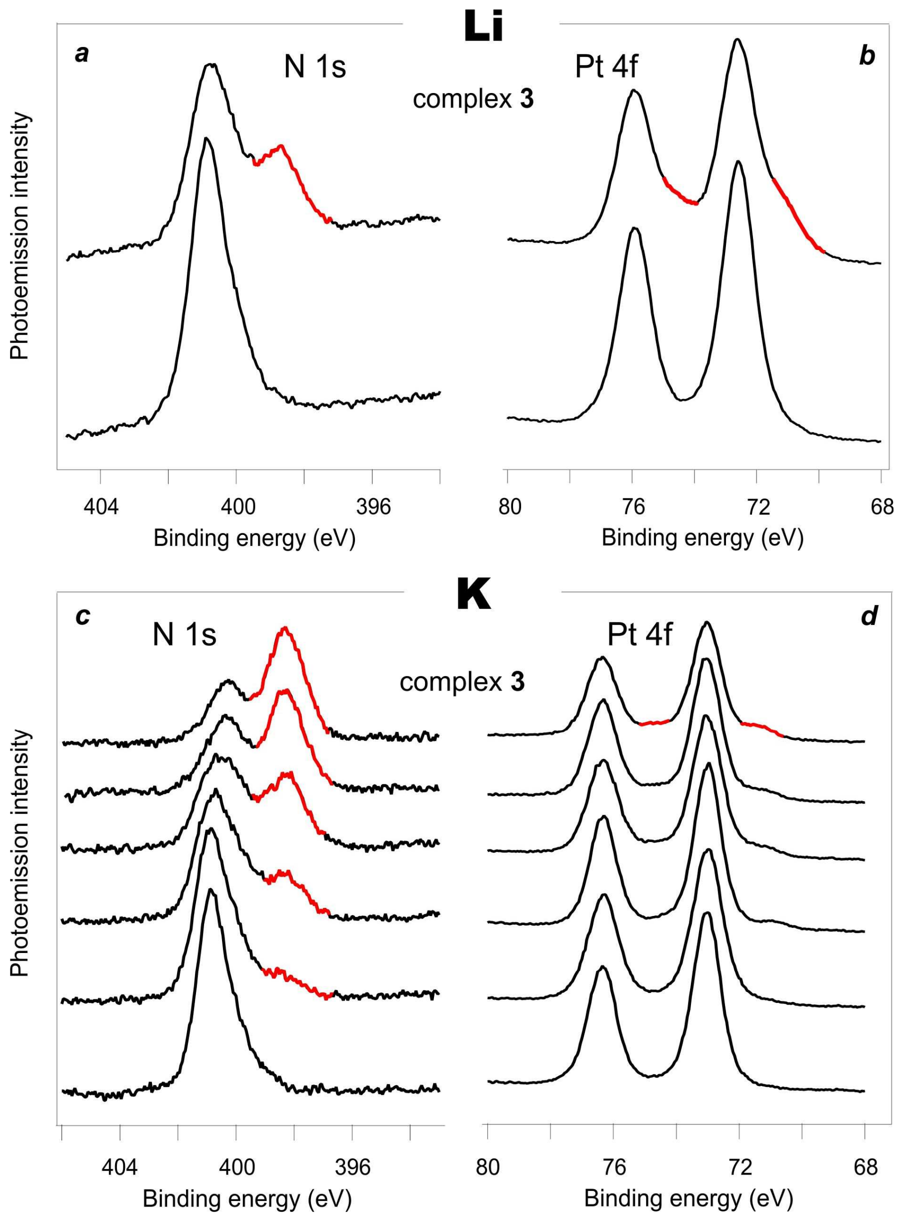

Figure S2. N 1s (a), (c) and Pt 4f (b), (d) core-level spectra of the pristine complex **3** and after deposition of Li and K, respectively.

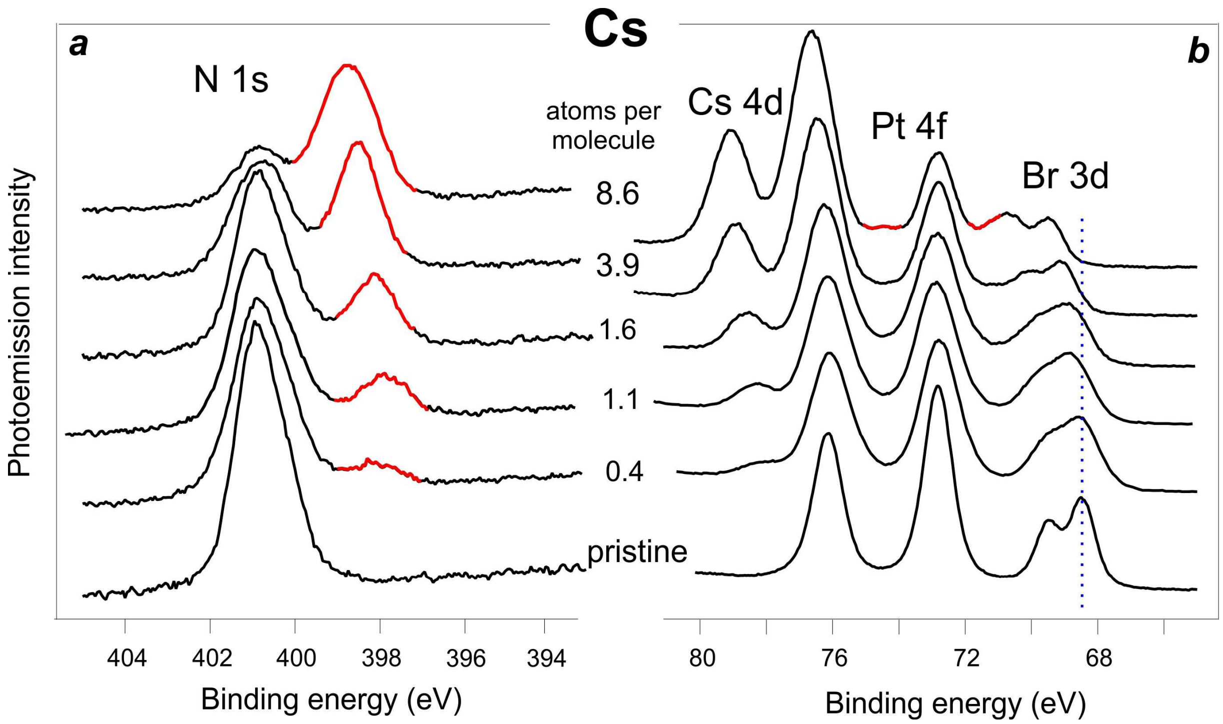

Figure S3. N 1s (a) and overlapping Cs 4d, Pt 4f and Br 3d (b) core-level spectra of the pristine complex **1** and after sequential deposition of Cs.

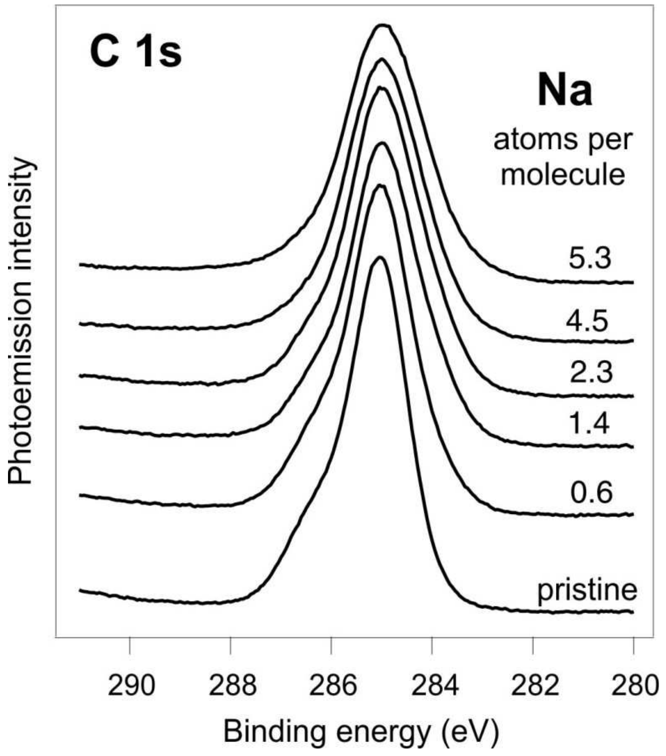

Figure S4. C 1s core-level PE spectra of the pristine complex **1** and after sequential deposition of Na.

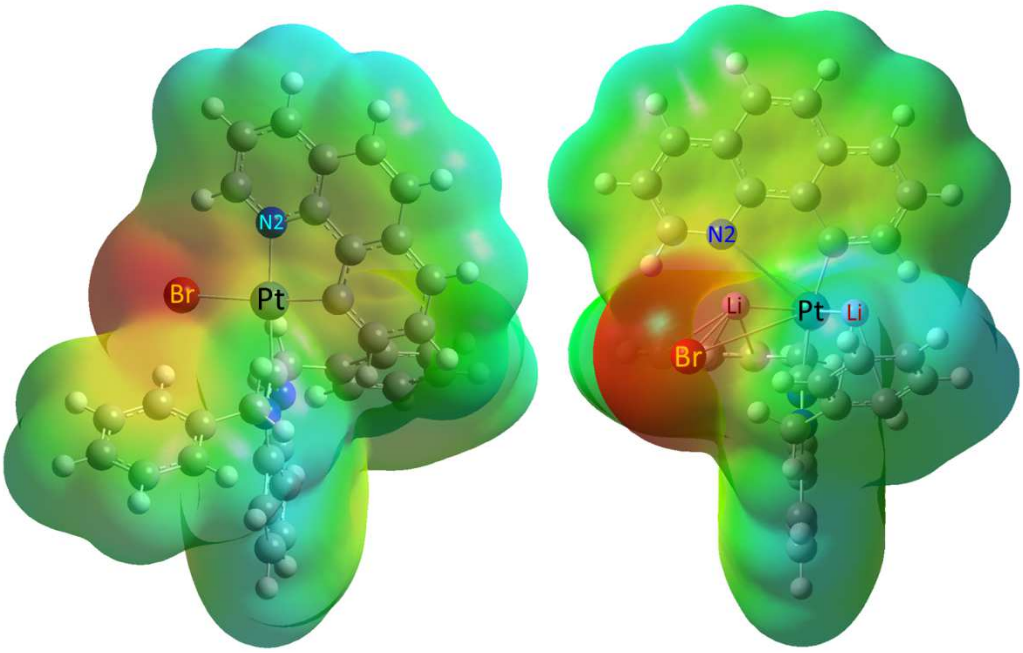

Figure S5. Mulliken charge distribution of the [Pt(N^C)(NHC)Br] molecule alone (left) and interacting with lithium atoms (right). The colour scale from red to blue, that represent negative and positive charges, respectively. Note the strong charge transfer interaction between Li and Pt atoms, and the visible electrostatic interaction between ionized lithium and electronegative bromine (on the right panel).

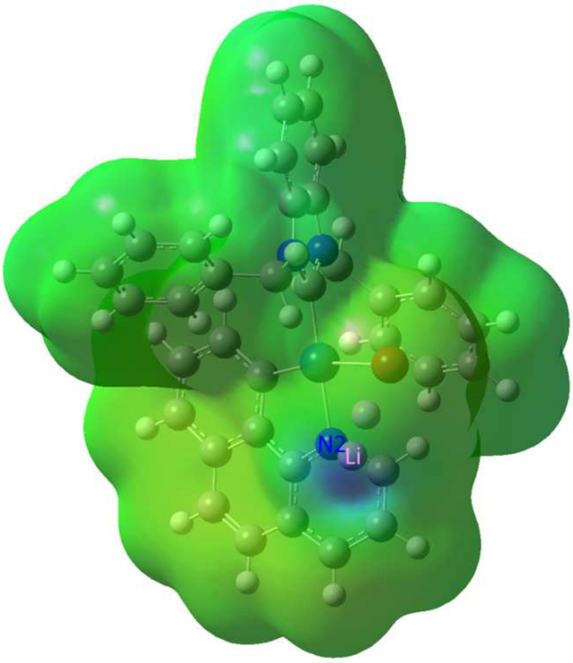

Figure S6. Mulliken charge distribution of the [Pt(N^C)(NHC)Br] molecule interacting with lithium atom – note the positive charge around the lithium atom as it donated electrons to the N2-atom the and Pt-centre.

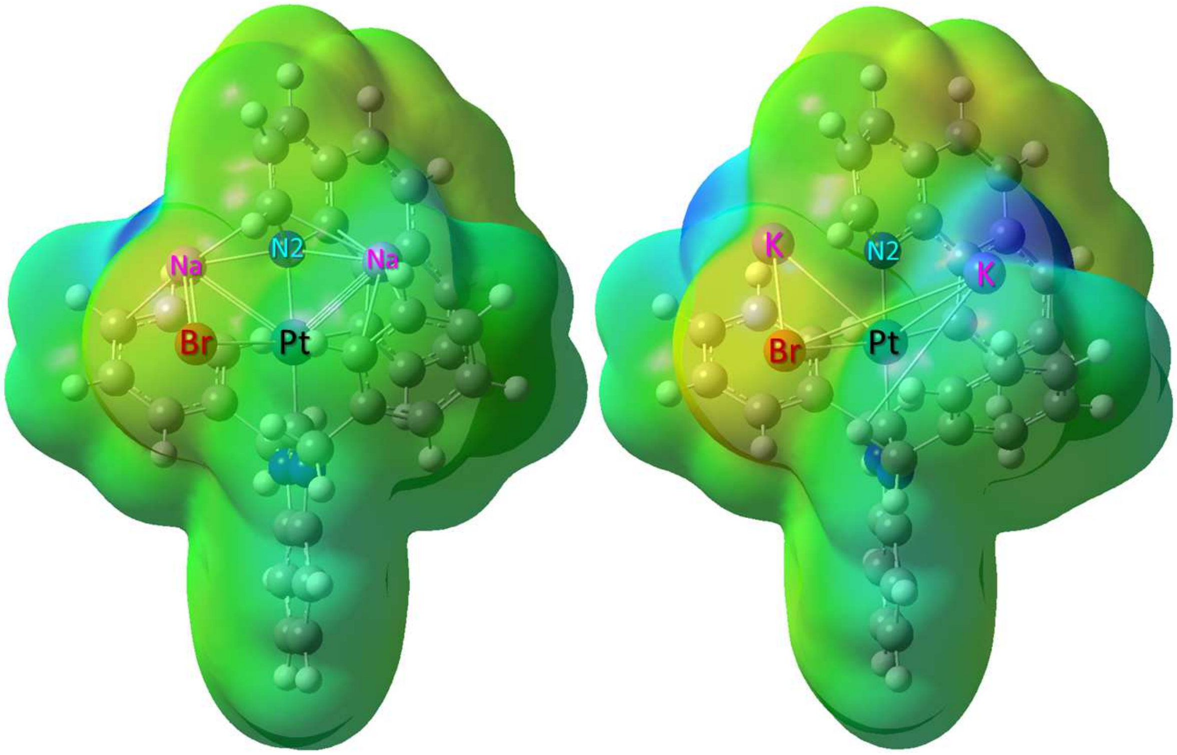

Figure S7. Mulliken charge distribution of the [Pt(N^C)(NHC)Br] molecule interacting with sodium (left) and potassium (right) atoms. The colour scale is the same as in Figure S5.
